# *Blechomonas campbelli* from *Ctenocephalides felis*: *in vitro* development, thermotolerance, and phylogenetic insights

**DOI:** 10.1590/0074-02760250286

**Published:** 2026-08-24

**Authors:** Letícia Gracielle Tôrres de Miranda Estevam, Alexei Yu Kostygov, Felipe Dutra-Rêgo, Anna Luiza Moreira Martins, Marina Andrade Freire, Ana Cristina Vianna Mariano da Rocha Lima, Daniel Moreira de Avelar, Gregório Guilherme Almeida, José Dilermando Andrade Filho, Vyacheslav Yurchenko, Gustavo Fontes Paz

**Affiliations:** 1 Fundação Oswaldo Cruz-Fiocruz, Instituto René Rachou, Belo Horizonte, MG, Brasil; 2 University of Ostrava, Faculty of Science, Life Science Research Centre, Ostrava, Czechia; 3 Russian Academy of Sciences, Zoological Institute, St. Petersburg, Russia; 4 Centro de Tecnologia de Vacinas, Belo Horizonte, MG, Brasil

**Keywords:** trypanosomatids, fleas, thermotolerance, morphology, phylogeny, host range

## Abstract

**BACKGROUND:**

*Blechomonas* spp. are flea-specific monoxenous trypanosomatids, whose biology, host range, and geographic distribution remain poorly understood, even for species inhabiting ubiquitous cat and dog fleas.

**OBJECTIVES:**

To isolate and characterise blechomonads from fleas of domestic dogs in Brazil, focusing on culture growth, morphology, and phylogenetic relationships.

**METHODS:**

Fleas collected from dogs in Sabará, Brazil, were morphologically identified and screened by cultivation. The isolate FL-1 was analysed for temperature-dependent growth, morphology, and phylogeny using 18S rRNA and gGAPDH sequences, including those mined from public metagenomic datasets.

**FINDINGS:**

Of 340 fleas (four per pool), only one culture tested positive. Growth was optimal at 25ºC, reduced at 30ºC, and absent at 35ºC. Four cell types were revealed, including a predominant “uromonad” stage specialised for attachment and aggregation. Phylogenetic analyses placed the isolate within *Blechomonas campbelli* and indicated that this species and *Blechomonas lauriereadi* occur in *Ctenocephalides* spp. from multiple continents.

**MAIN CONCLUSIONS:**

*Blechomonas campbelli* exhibits developmental adaptations to the flea gut and limited tolerance to elevated temperatures, constraining its potential to persist in warm-blooded hosts. These findings highlight the value of combining culture-based and *in silico* approaches to investigate flea-associated trypanosomatids.

Fleas (Insecta: Siphonaptera) are obligate haematophagous insects that parasitise a wide range of mammals, especially rodents in sylvatic environments, but also domestic animals, such as dogs and cats.^1^ The importance of fleas in animal and human health stems primarily from their role as vectors of epidemiologically significant pathogens, including *Yersinia pestis*, *Rickettsia* spp., *Bartonella* spp., and several haemoplasmas.^2^

Among microorganisms inhabiting fleas, monoxenous trypanosomatids of the genus *Blechomonas* Votýpka and Suková, 2013 are particularly notable, as they are strictly associated with these insects.^3^ The genus was formally described a little over a decade ago, when integrative morphological and phylogenetic analyses revealed that several flea-associated flagellate species form a distinct, deeply branching clade within the family Trypanosomatidae.^3^ Such a phylogenetic position justified assigning this lineage to a separate subfamily, Blechomonadinae Votýpka and Suková, 2013.^3^ It represents the third earliest-branching group among trypanosomatids, following the subfamilies Paratrypanosomatinae (*Paratrypanosoma* spp.) and Trypanosomatinae (*Trypanosoma* spp.), as confirmed by phylogenomic analyses.^4^ ^,^ ^5^

Despite recent advances, including the discovery of RNA viruses in some *Blechomonas* spp.^6^ and the genomic characterisation of *B. ayalai*,^7^ the life cycle, host range, and geographic distribution of these trypanosomatids are still poorly understood. Moreover, the number and geographic coverage of available isolates remain limited, although fleas are abundant, diverse, and cosmopolitan insects.^8^ Yet, *Blechomonas* represents a highly unusual lineage: unlike most monoxenous trypanosomatids that parasitise dipterans or true bugs, it is strictly associated with fleas. Along with its deep phylogenetic position, this suggests that its evolution was shaped by distinctive pressures and adaptations warranting detailed study.

While monoxenous trypanosomatids are arguably of lesser practical importance than dixenous ones, their complex evolutionary history, remarkable taxonomic diversity, and striking adaptive traits have attracted increasing attention in recent decades.^9^ ^,^ ^10^ ^,^ ^11^ In addition, monoxenous trypanosomatids have repeatedly been detected in vertebrate hosts, including humans, by polymerase chain reaction (PCR) or *in vitro* cultivation.^12^ In an early report, a parasite causing leishmaniasis-like symptoms in a human immunodeficiency virus (HIV)-infected patient showed kinetoplast DNA cross-hybridisation with a monoxenous species now placed in the genus *Blechomonas*,^3^ ^,^ ^13^ although the significance of this finding remains uncertain. Theoretically, blechomonads may be predisposed to colonise vertebrate hosts, given their specific association with fleas, haematophagous insects that transmit rodent trypanosomes of the subgenus *Herpetosoma*.^14^

In this study, we characterise the first Neotropical isolate of *Blechomonas campbelli* from fleas collected in south-eastern Brazil. Previously, another species of the same genus, *Blechomonas lauriereadi*, was reported from *Ctenocephalides felis* in localities of the Neotropical-Nearctic transition in Mexico.^15^ However, that study relied exclusively on molecular detection and did not establish parasite cultures suitable for experimental research. By combining *in vitro* cultivation, morphological analyses, and phylogenetic inference, we provide novel insights into the diversity, distribution, and biology of flea-associated trypanosomatids. In addition, we establish a cultured reference isolate to support future comparative and experimental studies.

## MATERIALS AND METHODS

*Sample collection and cultivation* - A total of 340 fleas were collected from 85 domestic dogs over an eight-month period (March to October 2019) in the municipality of Sabará, an endemic area for visceral leishmaniasis (VL) in the State of Minas Gerais, Brazil.^16^ Visual inspection of insects at the time of collection revealed no signs of recent blood feeding (*e.g.*, abdominal distension or visible blood); therefore, all specimens were considered unfed.

After collection, fleas were maintained in transport containers under controlled conditions (approximately 25ºC and 70-80% relative humidity) and processed on the same day at Fiocruz Minas (Belo Horizonte, Brazil). Prior to processing, specimens were briefly maintained in saline solution supplemented with penicillin (5,000 U/mL), streptomycin (500 μg/mL), and 5-fluorocytosine (50 μg/mL) to reduce microbial load.

Fleas were identified under stereomicroscopy using a taxonomic key for Brazilian species,^17^ based on diagnostic morphological characters including the shape of the frons, the relative length of the first spine of the genal comb, and the number of setae on the metepisternum (one-two setae in *C. felis* and three in *C. canis*). Fleas were then grouped into 85 pools (four individuals from the same host per pool) and mechanically macerated using sterile pestles for culture inoculation.

Primary cultures were established based on previously described protocols for the isolation of trypanosomatids,^18^ ^,^ ^19^ with minor modifications. Flea pools were incubated for 48 h at 4-8ºC in 1.5 mL tubes containing 1× phosphate-buffered saline (PBS) supplemented with antibiotics and antifungal agents to suppress microbial growth associated with the flea microbiota.^20^ Subsequently, samples were inoculated into biphasic Novy-MacNeal-Nicolle/Liver Infusion Tryptose (NNN/LIT) medium with the same antimicrobial supplementation and incubated at 25ºC, with weekly monitoring for the presence of flagellates. If trypanosomatids were detected, sub-culturing in the same medium was performed once per week.

For growth curve analysis, we adapted a previously described procedure for the thermotolerant trypanosomatid *Leptomonas seymouri*.^21^ Specifically, 5×10⁶ trypanosomatid cells were inoculated into 5 mL of biphasic medium and incubated for 14 days at three temperatures (25ºC, 30ºC, and 35ºC). Parasite concentrations were determined daily by diluting culture aliquots in 1× PBS containing 2% paraformaldehyde for fixation and counting cells in a Neubauer chamber, using technical triplicates and biological duplicates. Growth curves were compared in GraphPad Prism 8.0 (GraphPad Software, Boston, USA) using multiple t-tests with Holm-Šidák correction. The overall comparison of the growth curves was performed by calculating the area under the curve (AUC) using Simpson’s rule implemented in the SciPy library for Python. The differences between the conditions were estimated by the two-tailed Welch t-test.

*Morphological analysis* - Cultured cells were washed with 1× PBS, smeared onto glass slides, fixed in methanol for 30 min, and stained with Giemsa. The smears were examined under a light microscope with a 100× oil immersion objective, and images were captured using an AxioCam MRc colour camera (Zeiss, Jena, Germany). Photographed cells were measured using Fiji v.1.54j,^22^ estimating six standard morphometric parameters (when available): cell length (excluding flagellum), cell width, nucleus length, distances from the anterior end to the nucleus and kinetoplast, and free flagellum length.

To assess whether the visually defined cell categories could be discriminated based on morphometric features, we applied a Linear Discriminant Analysis (LDA) using the scikit-learn v.0.21.3 library in Python.^23^ Prior to analysis, missing values were imputed with the mean value within each cell category, while free flagellum length was set to zero for cells lacking a typical whip-like flagellum. All features were then standardised using the RobustScaler method to achieve zero median and unit variance. Since there were four cell types, up to k - 1 = 3 linear discriminants were retained. The model was trained on the full dataset without dimensionality reduction, and results were visualised using all pairwise combinations of the three linear discriminant axes (LD1-LD3) with the Python seaborn library v.0.9.0.^24^ Feature importance for each linear discriminant (defined as the absolute value of the corresponding LDA coefficient) was used to estimate the relative contribution of each morphometric measurement to cell type discrimination.

*DNA isolation, PCR, and sequencing* - Cultured cells were washed three times with 10 mL of 1× PBS by centrifugation at 3,000 rpm for 10 min at 4ºC. The resulting pellets were then used for DNA extraction with the PureLink^®^ Genomic DNA Mini Kit (Thermo Fisher Scientific, Waltham, USA).

The full-length (~2,100 bp) 18S rRNA gene was amplified using primers S762 (5’-GACTTTTGCTTCCTCTAWTG-3’) and S763 (5’-CATATGCTTGTTTCAAGGAC-3’),^25^ and subsequently sequenced with primers 883F (5′-GACTTGAATTAGMAAGCATGGGA-3′), 907R (5′-TCCCATGCTTKCTAATTCAAGTC-3′), A757 (5′-GCGAACGTACTCCCCCCTGA-3′), and S757 (5′-TCAGGGGGGAGTACGTTCGC-3′).^26^ Partial gGAPDH gene (~750-800 bp) was amplified and sequenced using the primers GAPtryF (5′-GGBCGCATGGTSTTCCAG-3′) and GAPtryR (5′-CCCCACTCGTTRTCRTACC-3′).^27^ PCR products were purified with the QIAquick^®^ PCR Purification Kit (Qiagen, Hilden, Germany) and sequenced by the Sanger method at the René Rachou Institute Sequencing Platform. The obtained sequences were deposited in GenBank under the accession numbers PX442322 and PX569296.

*Phylogenetic analyses* - The sequences generated in this study were combined with those available in the core nucleotide database of GenBank for *Blechomonas* spp. and four outgroup taxa. Additional sequences were obtained as follows: (i) sequence read archives (SRAs) of *Ctenocephalides* spp. containing trypanosomatid signatures were identified by BLASTn using the 18S rRNA sequence of *B. campbelli* (KF054134) as a query; (ii) reads from positive SRAs were assembled into the 18S rRNA and gGAPDH gene sequences in Geneious Prime 2024.0 as described previously.^28^ For both genes, sequences were aligned using MAFFT v.7.490 with the E-INS-i algorithm.^29^

The alignment of the 18S rRNA gene was trimmed twice: (i) low-quality ends of short sequences from GenBank identified by significant divergence from the conserved consensus for *Blechomonas* spp. were manually removed; (ii) the entire alignment was processed with trimAl v.1.5 using a gap threshold of 0.5.^30^ Maximum likelihood (ML) analysis was conducted in IQ-TREE v.2.3.6^31^ under the automatically selected TNe+I+G4 model with edge support assessed by both ultrafast and standard bootstrap methods (1,000 replicates each). Bayesian inference was performed in MrBayes v.3.2.7 under the GTR + I + G model, with 1,000,000 generations, sampling frequency of 100 and other parameters set to default.^32^

The analysis of the gGAPDH gene was performed similarly, but the alignment was not trimmed and partitioned by codon position as described previously.^33^ Briefly, analyses were conducted under partitioned models with linked branch lengths and unlinked evolutionary rates: TIM+F+G4/TPM3+G4/GTR+F+G4 in IQ-TREE and GTR+G+I in MrBayes.

*Ethics* - The collection of biological material from ectoparasites was conducted in accordance with Brazilian regulations. The study was approved by the Animal Use Ethics Committee (CEUA) of the René Rachou Institute - FIOCRUZ Minas under license number LW-14/17 and complies with Federal Law No. 11.794/2008 and the principles of the Brazilian Society of Laboratory Animal Science (SBCAL).

## RESULTS AND DISCUSSION

**Fig. 1:**
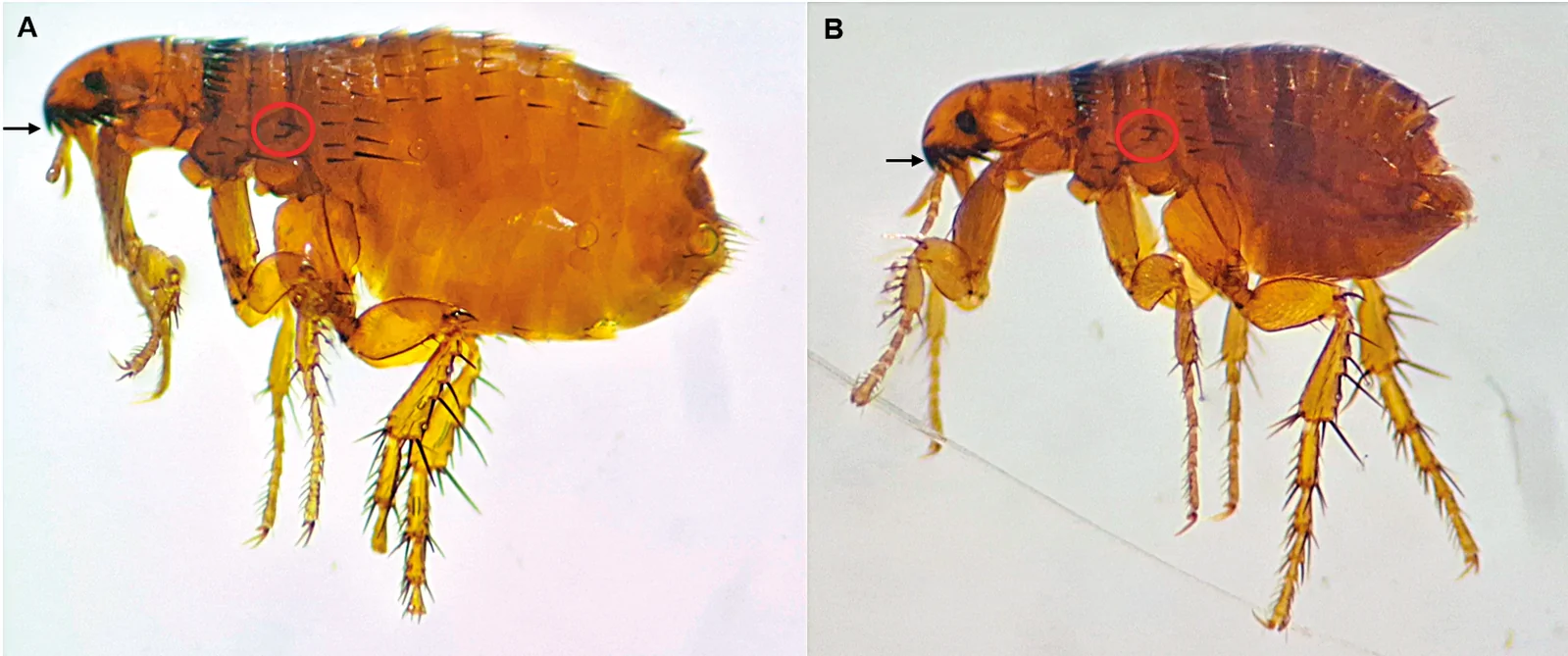
representative specimens of *Ctenocephalides felis*: female (A) and male (B). Diagnostic morphological characters include an elongated and anteriorly inclined frons, the genal comb with the first spine of similar length to the others (arrow), and the number of setae on the metepisternum (1-2 setae; red circle). Total magnification 350×.

All collected fleas were identified as *C. felis* (Fig. 1). Only one pooled sample (containing four fleas from a single dog) tested positive by cultivation. The obtained culture (isolate FL-1) was stable, which allowed us to study cell morphology.

**Fig. 2:**
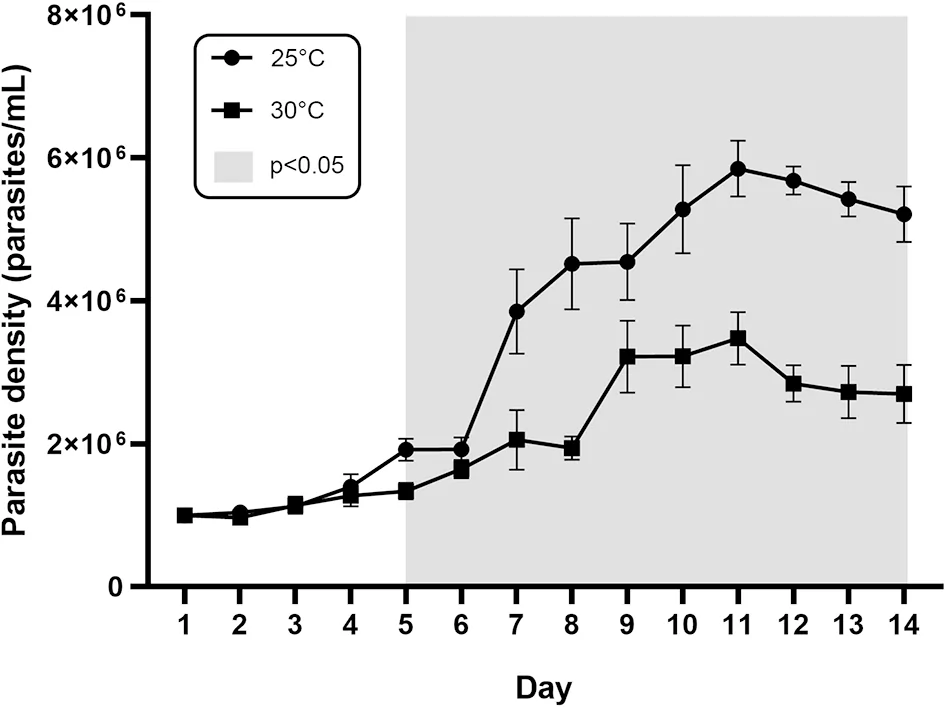
growth kinetics of the parasite in NNN/LIT biphasic medium at 25ºC and 30ºC. The grey shaded area marks time points with statistically significant differences in parasite concentrations between the two conditions (p < 0.05, multiple t-tests with Holm-Šidák correction).

*Analysis of parasite’s thermotolerance* - The growth of isolate FL-1 was temperature-dependent. At 25ºC, a brief lag (days 1-3) was followed by exponential growth until day 7, a plateau at ~5.8× 10⁶ parasites/ml (days 8-10), and a gradual decline. Growth at 30ºC was slower, with significant differences from day 5 onward, ~1.7-fold lower peak densities, and a shorter stationary phase, indicating moderate thermal stress (Fig. 2). The overall comparison of the curves using the AUC analysis showed their highly significant difference (p = 2×10⁻⁸) in growth dynamics between 25 and 30°C [Supplementary data (Table)]. At 35ºC, no proliferation was observed, and all cells died after three days. Together with other lines of evidence, including the absence of recent blood feeding at the time of collection and the known association of other *Blechomonas* spp. with fleas, this supports the interpretation that the isolate FL-1 is a genuine flea parasite rather than one derived from a transient blood meal.

These results are consistent with observations in other trypanosomatids, where temperatures slightly above the optimum trigger stress responses, increased cell death,^34^ or abrupt reductions in parasite load.^35^ Thermotolerance in monoxenous species has been associated with the upregulation of protective genes (*e.g.*, desaturases, chaperones, flagellar proteins) at elevated temperatures.^21^ ^,^ ^36^ However, addressing the molecular basis of the observed temperature-dependent growth patterns in the studied strain is beyond the scope of the current work.

**Fig. 3:**
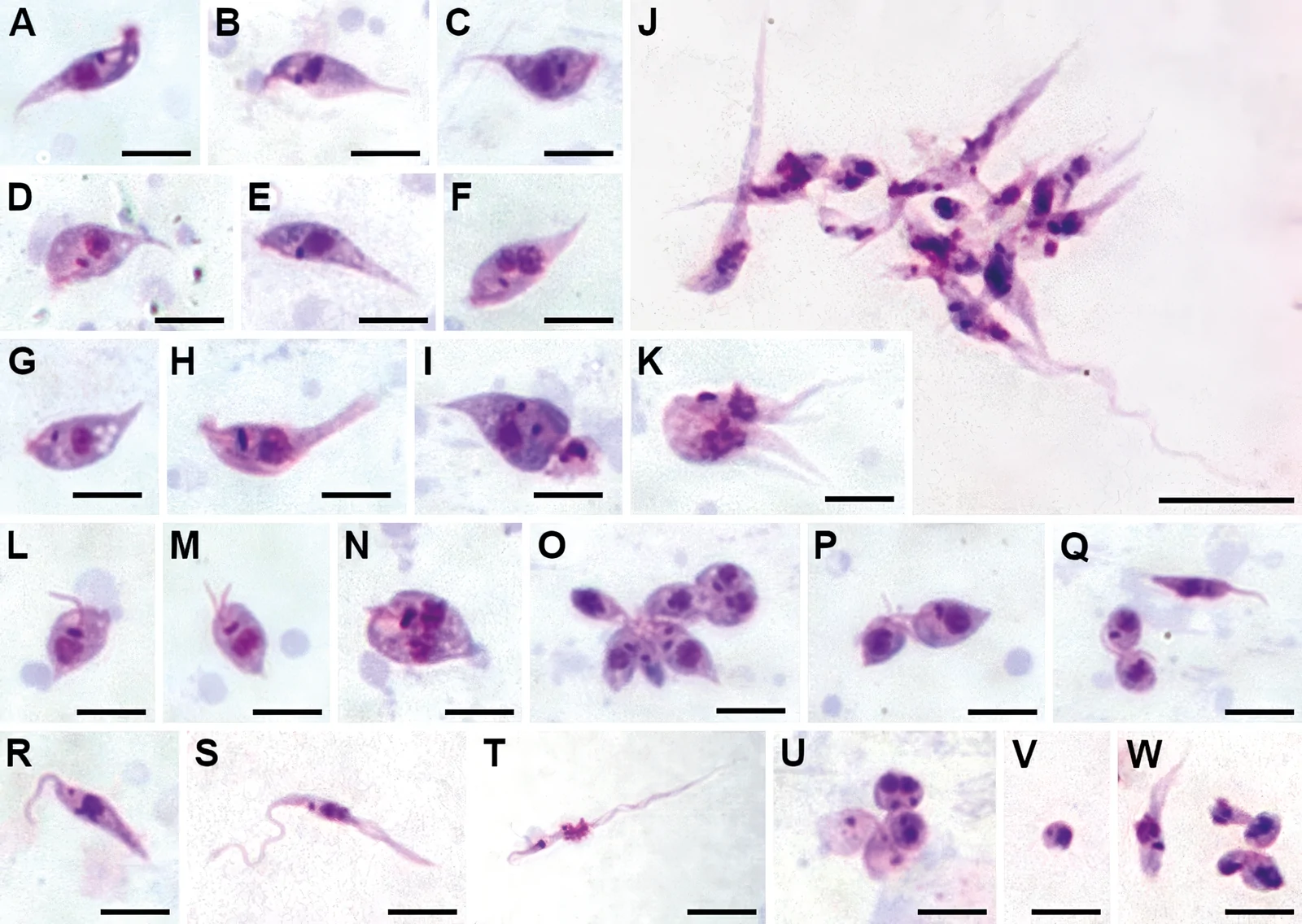
morphology of cells in the culture. (A-G) single uromonads; (H) early division of uromonad (two flagella and widened kinetoplast are visible in the cell); (I) asymmetric division of uromonad-like cell; (J) heterogeneous rosette of uromonads and promastigote-like cells; (K) uromonads attached to a matrix-like substance; (L) single choanomastigote; (M-N) dividing choanomastigotes; (O-P) rosettes of choanomastigotes of different sizes and shapes; (Q) pair of joined choanomastigotes (left) and short promastigote (right); (R-T) promastigotes; (U-V) amastigotes; (W) narrow uromonad (left) and group of amastigotes (right). Scale bars: 5 μm (A-I and K-W) and 10 μm (J).

*Morphological analysis* - Only a small proportion of cells in the culture swam freely, whereas the vast majority adhered to the plastic bottom, gradually forming large aggregates, which is a common strategy for many trypanosomatid species.^9^ In older cultures, these aggregates could detach from the substrate and float in the medium. The cells exhibited considerable variability in both shape and size (Table, Fig. 3). We tentatively classified them into four types: tailed cells (uromonads), choanomastigotes, promastigotes, and amastigotes.

**TABLE:** Morphometry of the four cell types observed in the culture

	Cell length	Cell width	Nucleus	A-N	A-K	Free flagellum	N
Amastigotes	2.5 ± 0.2 (2.2 - 2.9)	1.6 ± 0.2 (1.2 - 2.0)	1.2 ± 0.2 (0.9 - 1.6)	1.2 ± 0.2 (0.9 - 1.7)	0.7 ± 0.2 (0.3 - 1.1)	-	25
Choanomastigotes	4.5 ± 0.9 (2.7 - 6.1)	3.1 ± 0.7 (1.6 - 4.4)	1.9 ± 0.3 (1.4 - 2.6)	1.9 ± 0.6 (0.8 - 3.6)	1.3 ± 0.5 (0.4 - 2.6)	2.6 ± 0.8 (1.0 - 4.5)	50
Uromonads	10.4 ± 1.6 (7.2 - 14.5)	3.1 ± 0.6 (1.7 - 4.4)	2.1 ± 0.4 (1.6 - 3.4)	3.1 ± 0.5 (2.1 - 4.6)	2.1 ± 0.4 (1.0 - 3.0)	-	60
Promastigotes	16.1 ± 4.1 (10.8 - 23.8)	1.7 ± 0.3 (1.0 - 2.3)	2.1 ± 0.6 (1.2 - 4.2)	4.5 ± 1.3 (2.7 - 8.7)	1.8 ± 0.3 (1.7 - 3.3)	9.1 ± 2.3 (5.5 - 14.2)	30

A-N is the distance from the anterior end of the cell to the nucleus; A-K is the distance from the anterior end of the cell to the kinetoplast. All measurements are in μm [M ± SD (min-max)].

The LDA revealed clear separation among the four cell categories in the three-dimensional discriminant space, achieving classification accuracy of 98% ± 1% across folds, which indicates strong discriminatory power based on the morphometric features. In particular, LD1 and LD2 accounted for the majority of between-class variance and effectively discriminated uromonads and promastigotes, while choanomastigotes and amastigotes were separated when using projections with LD3 [Supplementary data (Figure A)]. The free flagellum length, distance from the anterior end to the kinetoplast, and the cell width were the most important features for the cell discrimination, contributing to LD1, LD2 and LD3, respectively [Supplementary data (Figure B)]. Of note, the culture also contained relatively rare intermediate forms, which were excluded from the analysis.

The predominant cell type was the tailed form, for which we propose the term “uromonad” (from Ancient Greek οὐρά — tail, and μονάς — unit). The name is reminiscent of terms such as necto- and haptomonad, which are used for developmental stages of *Leishmania* spp. in the sand fly gut.^37^ These cells were characterised by an elongated body with a clear contrast between its main swollen portion and a significantly narrower “tail”, creating a tadpole-like appearance (Fig. 3A-K). The free flagellum was either not detectable or appeared as a short, wide, and blurry structure, suggesting that it might be transformed into an attachment organelle characteristic of various trypanosomatids.^9^ The cells were proliferating at this stage (Fig. 3H-I). The presence of uromonads with two nuclei but no signs of duplication of other organelles suggests that cell division may be prone to aberrations. Although their flagellum was reduced, these attached cells were still capable of slight waggling movements. When aggregated, they could produce an amorphous matrix that covered the anterior portion of the cell (Fig. 3K), a feature especially apparent in old cultures. This trait is reminiscent of the behaviour observed in some other trypanosomatids, such as *Trypanosoma theileri* and *Lotmaria passim*, which secrete protective biofilms when attaching to the insect hindgut.^38^ ^,^ ^39^

Choanomastigotes were shorter than uromonads and when their posterior end was pointed, it was not distinctly separated from the main part of the cell. As a result, they generally appeared drop-shaped (Fig. 3L-P). Oval cells lacking posterior tapering appeared smaller (Fig. 3Q). Choanomastigotes possessed a flagellum that was typically shorter than the cell body. Nevertheless, it appeared to facilitate aggregation of cells into rosettes (Fig. 3O-Q). This cell type was also proliferative (Fig. 3M-N) and the presence of intermediate forms (Fig. 3I) suggests a developmental transition between choanomastigotes and uromonads.

Promastigotes represented another type of flagellated cells, ranging from relatively short, thick, club-shaped forms (Fig. 3Q-R) to very long cells with the body twisted along its axis and both ends tapered (Fig. 3S-T). In the latter case, they resembled the characteristic haemocoelic forms of *Phytomonas* spp.^40^ ^,^ ^41^ The flagellum in these cells varied in length but was always shorter than the cell body. Despite their evident motility, these cells were also observed in rosettes, where they appeared to originate from elongated uromonads (Fig. 3J).

Amastigotes were the typical oval to rounded aflagellate cells, either comparable in size to oval choanomastigotes or smaller (Fig. 3U-W). We hypothesise that amastigotes and oval choanomastigotes may transform into each other. No intracellular portion of the flagellum could be observed in light microscopy images; therefore, we refer to these cells here as amastigotes rather than endomastigotes, which are known from the life cycles of various monoxenous trypanosomatids and phytomonads and primarily serve for transmission.^9^

The observed diversity of cell types suggests a relatively complex life cycle that appears to be reproduced in culture. The ability of most cell types to attach, especially pronounced in uromonads, is likely crucial for survival in fleas, which can pump blood through their intestine in volumes exceeding 15 times their body volume per day.^42^ The fibrous matrix produced by these cells apparently helps them to hold on the hindgut surface, a site from which the majority of species were reported.^3^ However, the anatomical localisation of the studied parasite within the flea was not assessed in this study (as isolation was performed from whole-body macerates), nor in the original description of the species,^3^ to which it belongs according to phylogenetic analysis (see below). Therefore, the proposed association of this trypanosomatid with the hindgut and the functions of the observed cell types should be considered inferential and warrant confirmation through future *in situ* investigations.

Amastigotes, the only cell type that is unable to attach, are likely to be effectively shed with faeces and serve to infect other individuals, presumably larvae, which readily feed on the adult flea droppings.^43^ The rapid drying of these droppings (known as flea dirt) likely forces parasites to develop elevated resistance to adverse environmental conditions, analogous to compact aflagellate stages of other trypanosomatids — endomastigotes and cyst-like amastigotes.^9^ Motile choanomastigotes and promastigotes are likely involved in spreading within the host. The reason for the coexistence of two such morphotypes is unclear, although the non-proliferating nature of promastigotes suggests that their formation may be triggered by specific conditions, such as host starvation, which is a known factor for cell differentiation in various trypanosomatids.^44^ ^,^ ^45^

Although our morphological analysis was not intended for taxonomic identification or revision, it appeared natural to compare obtained our data with those available in the literature. The original description of *B. campbelli* (to which the FL-1 strain was eventually assigned, see below) is extremely brief, reporting “bended” promastigotes (subdivided into short- and long-flagellated forms) as the main morphotype, with only three measurements, and rare (unmeasured) amastigotes.^3^ Given the limited information and the absence of illustrations, a reliable morphological comparison was not possible. Therefore, our taxonomic assignment of the FL-1 strain was based exclusively on molecular data. We, nevertheless, speculate that promastigotes with short flagella may correspond to uromonads, whereas those with long flagella likely correspond to the promastigotes identified in our study. The reported size ranges considerably overlap with our measurements.

Interestingly, an earlier study of trypanosomatids isolated into culture from the gut of a cat flea provided a more detailed morphometric analysis, distinguishing four cell morphotypes with measurements largely overlapping those of strain FL-1, although also without illustrations, which complicates direct comparison.^46^ The main differences from our data concern the upper limits of promastigote width (6.2 vs 2.3 μm) and flagellum length (24.9 vs 14.2 μm). Although the maximal length and width of “cystic-like bodies” are bigger than those of FL-1 amastigotes (6.4 vs 2.9 μm and 3.8 vs 2.0 μm, respectively), this may reflect inclusion of choanomastigotes, which fall within these size ranges (referred to as “rounded amastigotes” in the cited study). We cannot determine whether the same or a different species was examined in that study, as the observed differences may reflect variation in culture conditions or strain-specific traits. The only molecular analysis reported in that paper excluded affiliation of the trypanosomatid with *Leishmania*, but did not resolve its taxonomic identity.^46^

**Fig. 4:**
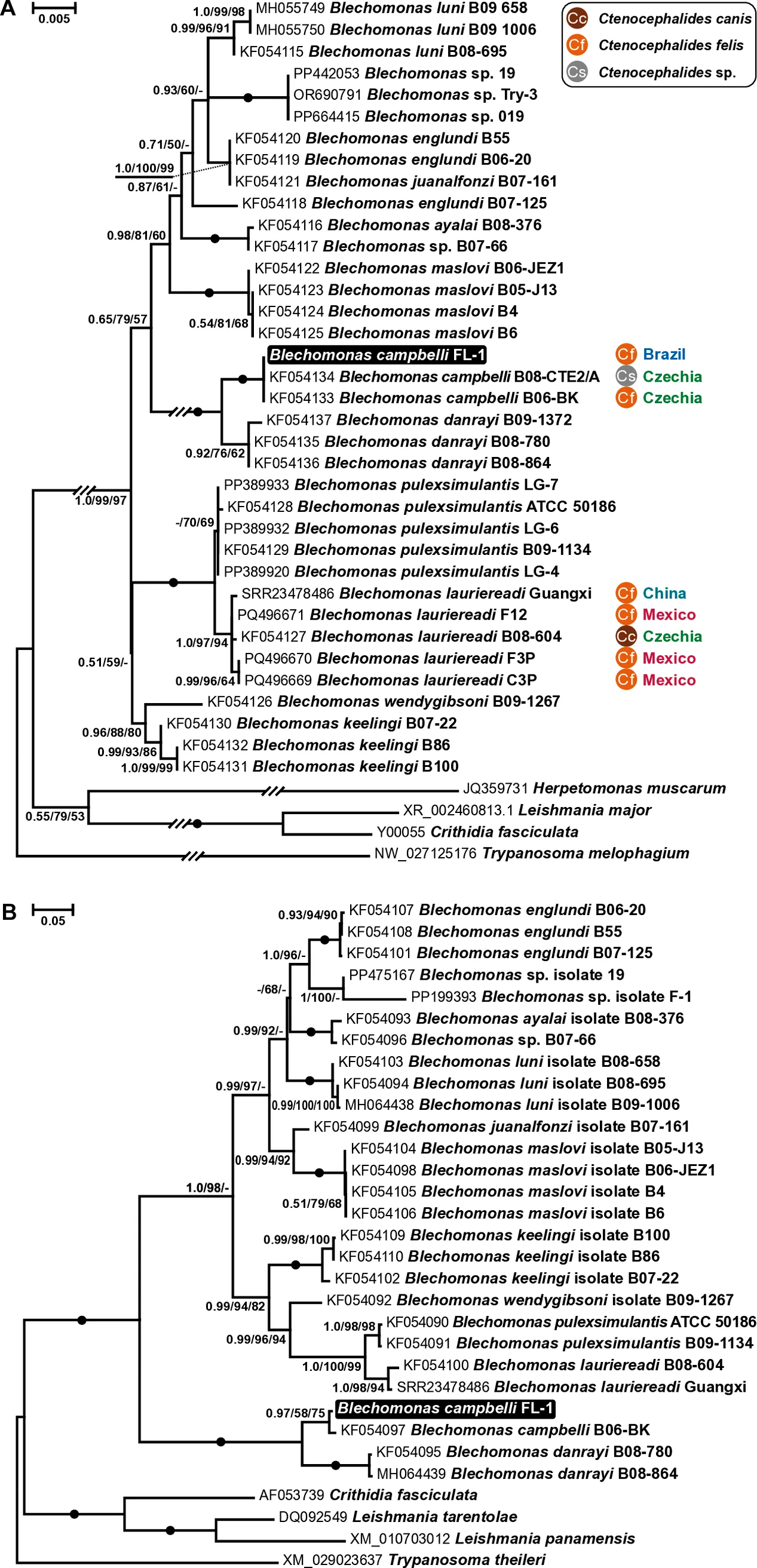
maximum likelihood phylogenetic trees of *Blechomonas* spp. based on two different genes. (A) 18S rRNA. (B) gGAPDH. Both trees are rooted using complex outgroups, with trypanosomes representing the earliest branch according to the phylogenomic inference.^(4)^ The isolate obtained in this study is highlighted in black. Geographic and host origins are indicated for *Blechomonas* spp. from cat and dog fleas. Numbers at branches are Bayesian posterior probabilities, as well as ultrafast and standard bootstrap supports (values below 0.5 or 50% are replaced with dashes or omitted). Black circles indicate maximum supports for all three methods (1.0/100/100). The triple-crossed branches were rescaled to one-third of their original length. The scale bar denotes the number of substitutions per site.

*Phylogenetic analyses* - The phylogenetic trees reconstructed from the 18S rRNA and gGAPDH gene sequences displayed different relationships among species (Fig. 4). Notably, the topology based on the latter gene was consistent with the tree inferred from the concatenated dataset of both genes, which was published in the study establishing the genus and describing most of its species.^3^ This suggests that in the concatenated dataset the overall topology was mainly determined by the gGAPDH gene. In our case, using the two genes separately was justified since the number of available sequences for them was significantly different and, moreover, they showed different variability levels.

The sequences of both genes from the studied isolate reliably affiliated it with *B. campbelli* (Fig. 4). While for the 18S rRNA gene there were no differences from the previously published sequences of that species, gGAPDH exhibited 10 substitutions at the 3’ end of the gene, yet all these were synonymous, *i.e.*, not reflected in the amino acid sequences. In sum, the sequence data support the assignment of the new isolate to *B. campbelli*.

Our analysis of SRAs from *Ctenocephalides* spp. revealed trypanosomatid reads in a metagenomic dataset of *C. felis* from Guangxi, China (GenBank accession number SRR23478486). We successfully assembled sequences of both genes despite the low read coverage for gGAPDH (4×). The sequences unambiguously clustered with *B. lauriereadi* (Fig. 4) and, relative to the previously analysed isolates of this species, differed by only 0-2 substitutions in the 18S rRNA gene and 16 substitutions (+1 N) in the gGAPDH gene, corresponding to a single amino acid change. These findings highlight the value of publicly available datasets for parasite surveillance, enabling detection of cryptic infections and expanding knowledge of parasite distribution and host associations with minimal additional sampling.

In total, the inferred 18S rRNA gene tree comprised eight sequences of flagellates from *Ctenocephalides* spp.: three belonged to *B. campbelli* and the other five to *B. lauriereadi* (Fig. 4A). Both species have been found in *C. felis*, but only a single isolate of the latter was from *C. canis*. Such a situation is likely explained by the fact that the first flea species is quite common, while the second is relatively rare.^47^ The clades of both trypanosomatid species included sequences from the New World (Brazil or Mexico) and Old World (Czechia and/or China). Therefore, although the currently available data remain limited, the results suggest that the same blechomonads can occur in different host species and geographic regions, indicating a lack of strict host or geographic association. This is apparently determined by the very wide specificity (euryxeny) of *C. felis*, which, despite its common name (cat flea), infests a broad range of unrelated hosts, including carnivores, ungulates, rodents, lagomorphs and marsupials.^43^ Ease of host switching, high mobility, and association with domestic animals in the era of globalisation likely facilitate genetic homogenisation of parasite populations across continents and host taxa. Although less is known about the dog fleas, both species definitely can coexist on the same host and, therefore, exchange parasites.^48^

Previous studies of trypanosomatids in fleas from Brazil, including those conducted in Minas Gerais, have reported the presence of trypanosomatids in *Ctenocephalides* spp. in the context of canine visceral leishmaniasis surveys.^46^ ^,^ ^49^ However, these studies lacked detailed morphological characterisation and molecular identification, limiting taxonomic resolution and comparability at the species level. In this context, the present study complements earlier findings by providing an integrative approach based on cultivation, microscopy, and sequence-based identification.

*In conclusion* - This study provides the first detailed analysis of the flea-associated trypanosomatid *B. campbelli*, the original description of which was limited to a brief diagnosis and its phylogenetic placement.^3^ The studied isolate displayed temperature-dependent growth: cells survived and proliferated at lower temperatures, but all died at 35ºC, which prevents sustained development in warm-blooded vertebrate hosts. Morphological analyses revealed a remarkable diversity of proliferative and non-proliferative cell types, including a predominant “uromonad” stage specialised for attachment and aggregate formation, likely representing an adaptation to survival in the flea gut. Phylogenetic analyses corroborated the identification and, together with mining publicly available sequencing datasets, expanded the known distribution of trypanosomatids associated with *Ctenocephalides* spp. (*B. campbelli* and *B. lauriereadi*) across continents and host species. These findings demonstrate that blechomonads are neither host- nor region-restricted, a pattern likely facilitated by the broad host range and global spread of *C. felis*. Our work highlights the value of combining culture-based studies with *in silico* surveillance for uncovering the diversity, biology, and epidemiology of flea-associated trypanosomatids.

## SUPPLEMENTARY MATERIALS

Supplementary material

## Data Availability

The contents underlying the research text are included in the manuscript.
