# Supplementary material for: Blechomonas campbelli from Ctenocephalides felis: in vitro development, thermotolerance, and phylogenetic insights
Source: Mem Inst Oswaldo Cruz. 2026 Aug 24;121:e250286. doi: 10.1590/0074-02760250286 (PMC13502892; doi:10.1590/0074-02760250286)
Supplement: Supplementary file 1 [file 1678-8060-mioc-121-e250286-s1.pdf]

TABLE

Raw statistical data for growth curves at 25ºC and 30ºC, including area under the curve (AUC) analysis

	25ºC (1st experiment)				25ºC (2nd experiment)					
	Sample 1	Sample 2	Sample 3	Mean (SD)	Sample 1	Sample 2	Sample 3	Mean (SD)	Overall mean	Overall SD
Day 1	1000000	1000000	1000000	1000000 (0)	1000000	1000000	1000000	1000000 (0)	1000000	0
Day 2	966666	1060000	1066666	1031111 (55910)	1173333	973333	1000000	1048889 (108594)	1040000	77860
Day 3	1073333	1166666	1093333	1111111 (49141)	1133333	1126666	1166666	1142222 (21430)	1126666	37947
Day 4	1140000	1520000	1566666	1408889 (234030)	1313333	1286666	1566666	1388888 (154536)	1398889	177710
Day 5	2033333	1746666	1700000	1826666 (180493)	2033333	2026667	1966667	2008889 (36717)	1917778	153401
Day 6	2026667	2126667	1966667	2040000 (80829)	1966667	1700000	1746666	1804444 (142413)	1922222	165445
Day 7	4266666	3666666	3233333	3722222 (518902)	3126667	4346667	4466667	3980000 (741440)	3851111	589518
Day 8	4400000	3950000	4183333	4177778 (225051)	4033333	5583333	4966667	4861111 (780373)	4519444	635559
Day 9	4466666	4466666	4883333	4605555 (240563)	3566667	4850000	5050000	4488889 (804904)	4547222	535144
Day 10	4500000	5533333	5216666	5083333 (529412)	4733333	5483333	6216667	5477778 (741683)	5280555	615487
Day 11	5516667	5933333	5583333	5677778 (223814)	6100000	5500000	6466667	6022222 (488004)	5850000	388444
Day 12	5833333	5816666	5883333	5844444 (34695)	5366667	5583333	5616666	5522222 (135742)	5683333	197484
Day 13	5366667	5583333	5216666	5388889 (184341)	5833333	5216666	5316667	5455555 (330964)	5422222	242365
Day 14	4950000	5950000	5316667	5405556 (505891)	4933333	5083333	5033333	5016666 (76376)	5211111	387394
AUC	44596942	46019440	44748885	45121755 (781120)	43436110	46727219	48586944	46250091 (2615798)	45685923	1829592
	30ºC (1st experiment)				30ºC (2nd experiment)					
Day 1	1000000	1000000	1000000	1000000 (0)	1000000	1000000	1000000	1000000 (0)	1000000	0
Day 2	1073333	900000	913333	962222 (96456)	1086667	873333	946667	968889 (108389)	965556	91837
Day 3	946666	1146667	1260000	1117778 (158652)	1080000	1146667	1260000	1162222 (91003)	1140000	118209
Day 4	1193333	1493333	1220000	1302222 (166043)	1160000	1413333	1146667	1240000 (150259)	1271111	145673
Day 5	1226667	1466667	1373333	1355556 (120984)	1266667	1466667	1220000	1317778 (131036)	1336667	114678
Day 6	1446667	1606667	1780000	1611111 (166711)	1666666	1611111	1780000	1685926 (86076)	1648519	125538
Day 7	1666667	1780000	2190000	1878889 (275325)	2000000	1878889	2820000	2232963 (511983)	2055926	415671
Day 8	1666667	1883333	2150000	1900000 (242097)	1933333	2033333	1966667	1977778 (50917)	1938889	162161
Day 9	2800000	2900000	3250000	2983333 (236291)	3566667	4033333	2766667	3455556 (640601)	3219445	503368
Day 10	2890000	3153333	3566667	3203333 (341093)	3583333	3583333	2566667	3244444 (586972)	3223889	429953
Day 11	2933333	3750000	3700000	3461111 (457752)	3100000	3583333	3783333	3488889 (351320)	3475000	365262
Day 12	2566667	2890000	2566667	2674445 (186676)	2900000	2890000	3244444	3011481 (201814)	2842963	253592
Day 13	2890000	2566667	2190000	2548889 (350338)	2566667	3244444	2890000	2900370 (339007)	2724630	363493
Day 14	2633333	2566667	2123333	2441111 (277215)	2900000	3353333	2606667	2953333 (376179)	2697222	407499
AUC	25144167	27311667	27720000	26725278 (1384420)	27839445	29914813	28213612	28655957 (1106137)	27690617	1540883

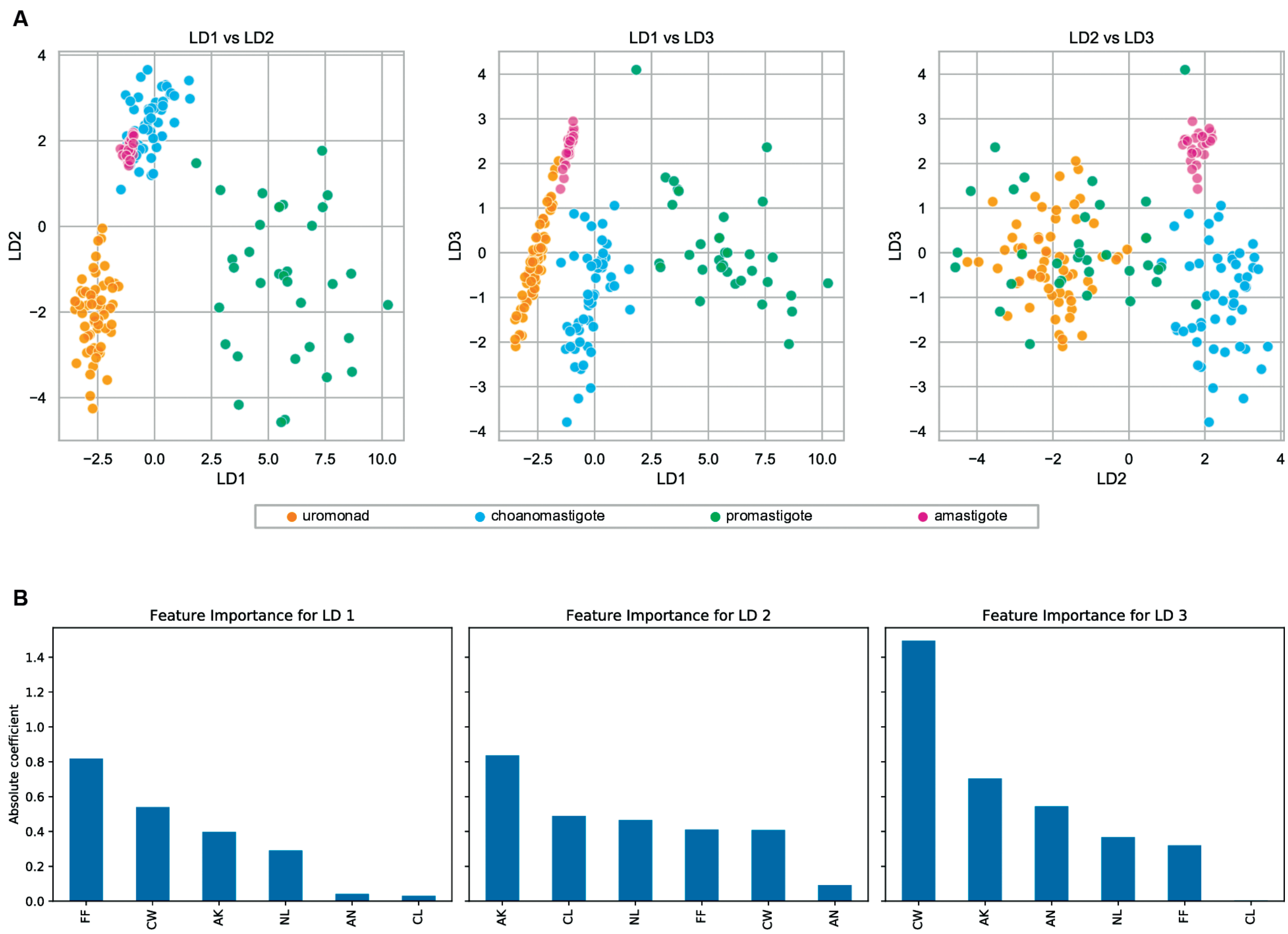

Linear discriminant analysis of morphometric data for four cell types. (A) Scatterplots showing pairwise projections of the first three linear discriminants. Each point represents a single cell, coloured by its assigned category (see graphical legend). (B) Bar plots showing the absolute feature contributions to linear discriminants (feature importance).
