# Supplementary material for: Blechomonas campbelli from Ctenocephalides felis: in vitro development, thermotolerance, and phylogenetic insights
Source: Mem Inst Oswaldo Cruz. 2026 Aug 24;121:e250286. doi: 10.1590/0074-02760250286 (PMC13502892; doi:10.1590/0074-02760250286)
Supplement: Peer review history

# Reviewer report: FIRST REVIEW ROUND - REVIEWERS COMMENTS

## REVIEWER #1

This study provides a valuable characterization of Blechomonas campbelli, combining morphological, thermotolerance, and phylogenetic analyses to expand knowledge of flea-associated trypanosomatids. However, several aspects of the methodology and data interpretation require clarification and cautious revision.

Comments:

1. The Introduction is concise, clearly written, and effectively establishes fleas as relevant vectors. The novelty of reporting the first Neotropical isolate is clearly stated, but the justification for its broader biological or epidemiological importance could be expanded. It could benefit from a slightly deeper discussion in the final paragraph to more explicitly define the research gap and the significance of obtaining a Neotropical isolate.

2. The sampling is limited to one municipality and one host (domestic dogs) only, so interpretations about “Neotropical isolates” should be cautious.

3. It is important to indicate that whether the fleas collected were fed or unfed, as this can strongly influence the understanding if the isolates were presence in blood meal (from the host) vs. true infection in the fleas. If both fed and unfed fleas were included, it would be important to indicate how pools were composed and whether feeding status was recorded or if there are any specific controls being included.

4. The authors employed 48 h incubation of flea pools at 4–8 °C prior to homogenization as a contamination control step. Although this may reduce contamination by other microbes, prolonged cold storage, however, also affect Blechomonas viability or it will allow the selection bias for more cold- or thermotolerant strains. Given that the later experiments were to assess the Blechomonas growth at different temperatures, this initial prolonged low-temperature incubation step may not fully represent the natural diversity of Blechomonas in the sample, and subsequent growth-temperature experiments could underestimate the species’ overall thermotolerance range. Please explained whether this protocol was adapted from a previous reference or if it has been validated to ensure parasite recovery and diversity were not compromised.

5. Please ensure that Genbank Accession Numbers are completed (Line 124: XXXXXX)

6. Line 158-159: Additional clarification for “No growth occurred at 35 °C” would be useful. Please specify if this is referred to absence of proliferation or cell death.

7. Line 163-164: The statement “the studied trypanosomatid may lack such mechanisms” appears to be speculative as there is no transcriptomic or genomic data were presented to assess gene presence or regulation. This inference should be removed or rephrased unless the authors provide molecular evidence to support this.

8. The conclusion that “limited thermotolerance excludes colonization of warm-blooded vertebrates” should therefore be stated more cautiously. This is because the 48-h cold incubation step before culture could have introduced selection bias before the isolation toward more cold-tolerant or less heat-tolerant strains. Unless additional data are available to demonstrate that other Blechomonas isolates similarly fail to grow at 35 °C or that cold incubation does not affect viability or diversity, this interpretation should be revised or rephrased to reflect the limitations of the current dataset.

## REVIEWER #2

Dear authors,

The manuscript provides valuable information on the association of Blechomonas with C. felis. It is written in a clear and fluid manner and contains all the essential elements expected for a study of this scope. However, several points remain overly general and would benefit from further clarification. Below are my detailed comments:

Line 43 – Such as?

Line 45 – Missing author and year.

Lines 48–49 – According to whom?

Line 52 – The phrasing here seems unnecessary. You may consider removing this sentence and introducing the findings in a different way.

Line 55 – Could you clarify what you meant by this?

Lines 78–86 – Is this protocol your own, or is it taken from the literature?

Lines 87–91 – Same question as above.

Line 124 – One sequence appears to be missing. Does this mean there was a single sequence for each gene?

Line 126 – Does this indicate something specific?

## REVIEWER #3

The abstract is well-written and summarizes the study’s objectives, methodology, and main findings. However, it would benefit from explicitly acknowledging key limitations, such as the low number of fleas analyzed per host and the identification of only one positive pooled sample as Blechomonas campbelli. Additionally, the abstract should address potential issues related to culture contamination, especially given the use of NNN/LIT medium. Including information on the health status of the sampled dogs would also enhance the contextual interpretation of the findings.

b) **Originality and Importance of the Contribution to the Field**

The manuscript tackles a relevant topic within vector biology, parasite surveillance, and biodiversity studies. The detection of a trypanosomatid in *Ctenocephalides felis* may be a significant observation. However, the originality and overall impact of the contribution are limited by the identification of only a single positive culture and by the absence of host-related contextual data, such as the clinical or health status of the dogs and the measurement of the parasite load in the infected flea. Without this information, the relevance of the findings for animal or public health remains uncertain.

c) **Relevance and Adequacy of Methodology, Results, and Discussion**

The methodology is clearly described and technically appropriate. However, the number of samples should be included in the methodology section rather than the results section. Flea collection, morphological identification, cultivation using NNN/LIT medium, and DNA analyses are all standard procedures. Nevertheless, analyzing only four fleas per dog, grouped into pooled samples, significantly limits the analysis of parasite’ thermotolerance, phylogenetic analyses, and for estimating infection frequency.

Moreover, the manuscript does not address the potential for contamination during culture, despite the known susceptibility of NNN/LIT medium to bacterial or fungal overgrowth, even when antifungal agents and antibiotics are used. The lack of information on contamination control measures and culture monitoring weakens the interpretation of the single positive result. Molecular confirmation through the amplification of trypanosomatid DNA from flea samples and of cultures is strongly recommended.

Additionally, the health status of the dogs (e.g., asymptomatic, clinically ill, or showing signs compatible with trypanosomatid infection, as well as serological results) is not reported. This information is essential for contextualizing the epidemiological and biological relevance of the findings and should be included and discussed, particularly regarding seroprevalence and the molecular characterization of *Leishmania* in dogs in the study area.

d) **References**

The references are mostly appropriate and generally up to date. They adequately support the study’s background; however, the authors may want to consider adding references on contamination culture risks in culture-based trypanosomatid studies and on methods for molecular confirmation.

e) **Figures and Tables**

The figures and tables are clear, well-organized, and properly labeled. They accurately reflect the sampling strategy and results. However, given the descriptive nature of the data, care should be taken to ensure that visual elements do not imply broader epidemiological conclusions.

## AUTHORS' RESPONSE TO THE REVIEWERS

January 29, 2026

Manuscript ID: MIOC-2025-0286

Blechomonas campbelli from Ctenocephalides felis - in vitro development, thermotolerance, and phylogenetic insights

Dear Dr. Dias-Lopes,

We sincerely thank you and the reviewers for the thorough and constructive evaluation of our manuscript. We carefully considered all comments and incorporated the suggested revisions to improve clarity, methodological transparency, and the overall scientific quality of the work. In several instances, the reviewers raised important conceptual and methodological points that helped us refine the presentation of our results and strengthen key interpretations.

Below, we provide a detailed, point-by-point response to all comments. Reviewer remarks are presented in italics, followed by our responses. For clarity, we refer to page and line numbers in the tracked-changes version of the manuscript. Whenever we slightly disagreed with a suggestion, we explain our reasoning while ensuring that the revised version adequately addresses the reviewer’s concern.

We hope that the revised manuscript meets the expectations of the editorial board and reviewers, and we remain at your disposal for any further adjustments if needed.

Sincerely,

Felipe Dutra Rêgo

Instituto René Rachou - Oswaldo Cruz Foundation

## Reviewer 1

Reviewer #1: This study provides a valuable characterization of Blechomonas campbelli, combining morphological, thermotolerance, and phylogenetic analyses to expand knowledge of flea-associated trypanosomatids. However, several aspects of the methodology and data interpretation require clarification and cautious revision.

1- The Introduction is concise, clearly written, and effectively establishes fleas as relevant vectors. The novelty of reporting the first Neotropical isolate is clearly stated, but the justification for its broader biological or epidemiological importance could be expanded. It could benefit from a slightly deeper discussion in the final paragraph to more explicitly define the research gap and the significance of obtaining a Neotropical isolate.

Authors’ comment: We expanded the final paragraph of the Introduction to better define the research gap and highlight the biological significance of obtaining a Neotropical isolate. Specifically, we now acknowledge previous molecular detections of Blechomonas in the region (i.e., B. lauriereadi in Ctenocephalides felis from Mexico), while emphasizing that this was not accompanied by isolation of cultures (lines 76–84).

2- The sampling is limited to one municipality and one host (domestic dogs) only, so interpretations about “Neotropical isolates” should be cautious.

Authors’ comment: We clarify that our statement refers specifically to B. campbelli, for which no previous isolates have been reported from the Neotropical region. Throughout the manuscript, we explicitly restrict our interpretation to fleas collected from domestic dogs in a single municipality and do not imply that this isolate is representative of the Neotropics as a whole.

3- It is important to indicate that whether the fleas collected were fed or unfed, as this can strongly influence the understanding if the isolates were presence in blood meal (from the host) vs. true infection in the fleas. If both fed and unfed fleas were included, it would be important to indicate how pools were composed and whether feeding status was recorded or if there are any specific controls being included.

Authors’ comment: None of the collected fleas showed visible signs of recent blood feeding at the time of sampling, and this information has been added to the Methods section (lines 89–91). Importantly, C. felis feeds frequently in small blood meals and rapidly defecates; therefore, the absence of visible blood does not necessarily indicate lack of recent feeding but rather reflects the feeding behavior of this species.

4- The authors employed 48 h incubation of flea pools at 4–8 °C prior to homogenization as a contamination control step. Although this may reduce contamination by other microbes, prolonged cold storage, however, also affect Blechomonas viability or it will allow the selection bias for more cold- or thermotolerant strains. Given that the later experiments were to assess the Blechomonas growth at different temperatures, this initial prolonged low-temperature incubation step may not fully represent the natural diversity of Blechomonas in the sample, and subsequent growth-temperature experiments could underestimate the species’ overall thermotolerance range. Please explained whether this protocol was adapted from a previous reference or if it has been validated to ensure parasite recovery and diversity were not compromised.

Authors’ comment: We thank the reviewer for this important methodological comment. The 48 h incubation at 4–8°C in antibiotic/antifungal-supplemented PBS was adopted as a contamination control step, given the well-documented richness and diversity of microbiota in C. felis. This rationale, along with the relevant references was added to the Methods section (lines 93–99).

Our previous experiments have shown that such incubation does not affect the viability of Leishmania. Moreover, this approach has been employed in published studies (e.g. Pereira et al., 2017, cited in the manuscript). Recommendations to maintain trypanosomatids at lowered temperatures (around 4°C or slightly higher) to avoid heat stress and/or suppress contaminant overgrowth are also included in the guidelines for “Field isolation and cultivation of trypanosomatids from insects” (cited in the manuscript) as well as in the WHO “Handbook on isolation, characterization and cryopreservation of Leishmania” (https://iris.who.int/handle/10665/60795). Taken together, these considerations indicate that the applied low-temperature incubation is a standard precautionary measure and is unlikely to have a significant impact on the intrinsic thermotolerance of tyrpanosomatids.

5- Please ensure that Genbank Accession Numbers are completed (Line 124: XXXXXX).

Authors’ comment: The actual GenBank accession numbers are now included in the manuscript.

6- Line 158-159: Additional clarification for “No growth occurred at 35 °C” would be useful. Please specify if this is referred to absence of proliferation or cell death.

Authors’ comment: We clarified this point in the Results section (line 180): “At 35°C, no proliferation was observed, and all cells died after three days”.

7- Line 163-164: The statement “the studied trypanosomatid may lack such mechanisms” appears to be speculative as there is no transcriptomic or genomic data were presented to assess gene presence or regulation. This inference should be removed or rephrased unless the authors provide molecular evidence to support this.

Authors’ comment: We rephrased this sentence as follows:

Whether these mechanisms in the studied trypanosomatid are absent or have limited efficiency is to be determined experimentally (lines 189–191).

8- The conclusion that “limited thermotolerance excludes colonization of warm-blooded vertebrates” should therefore be stated more cautiously. This is because the 48-h cold incubation step before culture could have introduced selection bias before the isolation toward more cold-tolerant or less heat-tolerant strains. Unless additional data are available to demonstrate that other Blechomonas isolates similarly fail to grow at 35 °C or that cold incubation does not affect viability or diversity, this interpretation should be revised or rephrased to reflect the limitations of the current dataset.

Authors’ comment: We respectfully maintain that the lack of parasite survival at 35°C provides strong biological evidence against adaptation to vertebrate body temperatures. However, to address the reviewer’s concern regarding potential selection bias during isolation, we rephrased the conclusion to avoid categorical wording. The revised text now refers only to the obtained isolate, rather than uncompromisingly “excluding” this possibility for any trypanosomatids inhabiting fleas (lines 294–297).

## Reviewer 2

Reviewer #2: The manuscript provides valuable information on the association of Blechomonas with C. felis. It is written in a clear and fluid manner and contains all the essential elements expected for a study of this scope. However, several points remain overly general and would benefit from further clarification.

1- Line 43 – Such as?

Authors’ comment: We specified examples of epidemiologically relevant pathogens transmitted by fleas (e.g., Yersinia pestis, Rickettsia spp., Bartonella spp.).

2- Line 45 – Missing author and year.

Authors’ comment: We added the taxon authorship information along with the appropriate reference (now citing reference 3; line 48).

3- Lines 48–49 – According to whom?

Authors’ comment: We added the taxon authorship information and the appropriate reference (lines 51-52).

4- Line 52 – The phrasing here seems unnecessary. You may consider removing this sentence and introducing the findings in a different way.

5- Line 55 – Could you clarify what you meant by this?

Authors’ comment: We rephrased the paragraph to improve clarity and flow (lines 55–66).

6- Lines 78–86 – Is this protocol your own, or is it taken from the literature?

7- Lines 87–91 – Same question as above.

Authors’ comment: The protocols were adapted from previous publications. We clarified this in the Methods and added supporting references (lines 93–105).

8- Line 124 – One sequence appears to be missing. Does this mean there was a single sequence for each gene?

Authors’ comment: We added the missing GenBank accession number.

9- Line 126 – Does this indicate something specific?

Authors’ comment:

The sentence was revised: “The sequences generated in this study were combined with those available in the core nucleotide database of GenBank...” (lines147-148).

## Reviewer 3

Reviewer #3:

1- Adequacy of the Abstract: The abstract is well-written and summarizes the study’s objectives, methodology, and main findings. However, it would benefit from explicitly acknowledging key limitations, such as the low number of fleas analyzed per host and the identification of only one positive pooled sample as Blechomonas campbelli. Additionally, the abstract should address potential issues related to culture contamination, especially given the use of NNN/LIT medium. Including information on the health status of the sampled dogs would also enhance the contextual interpretation of the findings.

Authors’ comment: We revised the Abstract to acknowledge key limitations, including the low number of fleas per pool and the recovery of a single positive culture. These points are now stated in the Findings and Main Conclusions sections. No issues related to culture contamination were detected, as no contaminating microorganisms were observed in any of the cultured samples. The single positive culture contained a trypanosomatid, whose presence is well supported by previous knowledge of flea-associated flagellates. The comment regarding the health status of dogs is addressed below.

2- Originality and Importance of the Contribution to the Field: The manuscript tackles a relevant topic within vector biology, parasite surveillance, and biodiversity studies. The detection of a trypanosomatid in Ctenocephalides felis may be a significant observation. However, the originality and overall impact of the contribution are limited by the identification of only a single positive culture and by the absence of host-related contextual data, such as the clinical or health status of the dogs and the measurement of the parasite load in the infected flea. Without this information, the relevance of the findings for animal or public health remains uncertain.

Authors’ comment: We acknowledge the limitations raised. However, this study was not designed as an epidemiological investigation. Its primary contribution consists in the isolation and biological characterization of B. campbelli, representing the first Neotropical isolate of this species and providing novel experimental data on its phenotype and phylogenetic placement. Importantly, in the context of this study, fleas - not dogs - are the hosts of the investigated trypanosomatids. We therefore consider the scope of the study and the conclusions drawn to be appropriate and believe that no changes are required.

3- Relevance and Adequacy of Methodology, Results, and Discussion:

a. The methodology is clearly described and technically appropriate. However, the number of samples should be included in the methodology section rather than the results section. Flea collection, morphological identification, cultivation using NNN/LIT medium, and DNA analyses are all standard procedures. Nevertheless, analyzing only four fleas per dog, grouped into pooled samples, significantly limits the analysis of parasite’ thermotolerance, phylogenetic analyses, and for estimating infection frequency.

Authors’ comment: We moved the sample size information (number of dogs and fleas) from Results to Methods (line 87).

b. Moreover, the manuscript does not address the potential for contamination during culture, despite the known susceptibility of NNN/LIT medium to bacterial or fungal overgrowth, even when antifungal agents and antibiotics are used. The lack of information on contamination control measures and culture monitoring weakens the interpretation of the single positive result. Molecular confirmation through the amplification of trypanosomatid DNA from flea samples and of cultures is strongly recommended.

Authors’ comment: Contamination control measures and culture monitoring are now explicitly described in the Methods. In addition, the isolate identity was molecularly confirmed using two independent markers (18S rRNA and gGAPDH genes). Direct molecular screening of flea pools was beyond the scope of this study and could not be reliably performed in parallel with culture-based detection due to the small body size of fleas.

c. Additionally, the health status of the dogs (e.g., asymptomatic, clinically ill, or showing signs compatible with trypanosomatid infection, as well as serological results) is not reported. This information is essential for contextualizing the epidemiological and biological relevance of the findings and should be included and discussed, particularly regarding seroprevalence and the molecular characterization of Leishmania in dogs in the study area.

Authors’ comment: Host clinical data were not collected, as sampling was designed exclusively for ectoparasites. This study did not aim to assess canine infection or public health relevance but was restricted to the characterization of flea-associated flagellates. Such data are therefore beyond the scope of the present work and are not required to support our conclusions.

4- References: The references are mostly appropriate and generally up to date. They adequately support the study’s background; however, the authors may want to consider adding references on contamination culture risks in culture-based trypanosomatid studies and on methods for molecular confirmation.

Authors’ comment: In response to a similar comment by another reviewer, we included two additional references supporting the culture procedure and contamination control measures. (see Methods).

5- Figures and Tables: The figures and tables are clear, well-organized, and properly labeled. They accurately reflect the sampling strategy and results. However, given the descriptive nature of the data, care should be taken to ensure that visual elements do not imply epidemiological conclusions.

Authors’ comment: We reviewed all figures and captions and confirm that they are strictly descriptive and do not imply epidemiological conclusions. No changes were required.

# Reviewer report: REVIEWERS COMMENTS

## REVIEWER #1

All suggestions have been accepted; therefore, I have no further comments, and the manuscript can be accepted in its current form.

## REVIEWER #2

Below are my comments in English, structured according to the requested evaluation criteria.

a) Adequacy of the Abstract – Adequate but Needs Greater Precision

The abstract is generally clear and aligned with the proposed objectives. It adequately summarizes the isolation, in vitro characterization, thermotolerance observations, and phylogenetic positioning of Blechomonas campbelli from Ctenocephalides felis.

The abstract would benefit from:

• Clearer specification of the methodological approaches used for host identification and parasite confirmation.

• More precise wording regarding the strength of the phylogenetic support.

• Avoiding overstatement of novelty without robust comparative evidence.

b) Originality and Importance of the Contribution

The isolation of Blechomonas campbelli from Ctenocephalides felis in a Neotropical region is potentially relevant, as flea-associated monoxenic trypanosomatids remain underexplored. Expanding geographic records contributes to understanding parasite diversity, distribution, and host associations.

However, the originality claim is weakened by:

• Insufficient comparison with previously described isolates.

• Lack of detailed morphometric comparison with the original description by Votypka & Suková (2013).

• Limited discussion of prior regional reports of monoxenic trypanosomatids in fleas (e.g., reports from Minas Gerais).

The study has potential importance for the field, but the current manuscript does not convincingly demonstrate a significant conceptual or methodological advance. Its contribution appears incremental rather than transformative.

c) Methodology, Results, and Discussion – Major Weaknesses

1. Host Identification – Methodological Deficiency

The identification of Ctenocephalides felis is not sufficiently documented.

• No detailed morphological description is provided.

• No indication of chemical clearing procedures for proper visualization of cephalic and comb structures.

• No molecular confirmation (e.g., COI barcoding) is reported.

• No explicit differentiation from Ctenocephalides canis or other related taxa.

Accurate host identification is essential in studies of monoxenic trypanosomatids. The lack of transparency compromises reproducibility and weakens ecological interpretation.

2. Isolation Procedures and Parasite Detection – Insufficient Detail

The manuscript does not provide adequate methodological details regarding:

• Time and conditions between flea capture and processing.

• Dissection procedures.

• Culture medium composition.

• Temperature conditions.

• Subculturing frequency.

• Contamination control measures.

Without these details, reproducibility is compromised. Isolation protocols should be described comprehensively, particularly in studies claiming a new geographic record.

3. Anatomical Localization – Missing Critical Information

The anatomical localization of the parasite within the flea is not clearly described.

Given that Blechomonas species are commonly associated with the hindgut surface, and the authors hypothesize the presence of a “uromonad” stage involved in attachment, the study should:

• Specify where parasites were observed.

• Provide microscopy images supporting localization.

• Compare findings with previous descriptions.

Localization is biologically relevant and cannot be omitted.

4. Morphological Characterization and Taxonomic Context – Incomplete Comparative Analysis

Although morphometric measurements were performed, there is no structured comparison with the original description of Blechomonas campbelli.

This is particularly important because:

• Species in this genus exhibit overlapping morphological traits.

• Morphology alone is insufficient for reliable identification.

• Previous cases (e.g., reclassification of Leptomonas pulexsimulantis to Blechomonas) demonstrate the limitations of morphology-based taxonomy.

A side-by-side morphometric comparison table would significantly strengthen the manuscript.

5. Phylogenetic Analyses – Limited Taxon Sampling

The phylogenetic analysis would benefit from:

• Broader inclusion of representative isolates from multiple trypanosomatid genera.

Restricted sampling can artificially inflate bootstrap or posterior probability values due to reduced topological alternatives. Strong support values under limited sampling do not necessarily reflect true phylogenetic stability.Broader sampling would improve clade stability and strengthen species-level assignment.

6. Functional Evidence for Thermal Adaptation – Unsupported Claims

The manuscript discusses thermotolerance but does not provide mechanistic or functional validation.

If heat tolerance is proposed as a biological trait, it should be supported by:

• Growth curves at multiple temperatures.

• Statistical comparison of proliferation rates.

• Gene expression assays (e.g., RT-qPCR) evaluating HSP70, HSP90, desaturases, and flagellar proteins.

• Normalization against housekeeping genes such as GAPDH.

The mention of multiple copies of HSP-related genes in the genome is insufficient to infer functional adaptation without expression data.

Currently, conclusions regarding thermal adaptation are speculative.

## REVIEWER #3

The authors have adequately addressed all the concerns raised during the first round of review. The revisions have improved the clarity and quality of the manuscript. I therefore support the acceptance of the manuscript.

## AUTHORS' RESPONSE TO THE REVIEWER

Manuscript ID: MIOC-2025-0286

*Blechomonas campbelli* from *Ctenocephalides felis*: *in vitro* development, thermotolerance, and phylogenetic insights

Dear Dr. Dias-Lopes,

We thank you and the reviewers for the careful evaluation of our revised manuscript.

Following the second round of review, two reviewers (Reviewers 1 and 3) recommended acceptance without further comments, while Reviewer 2 provided several minor suggestions. All comments have been carefully addressed point by point, with revisions made to improve clarity, methodological transparency, and interpretation. Where suggestions extended beyond the scope of the study, we clarified the limitations and adjusted the text accordingly.

Below, we provide a detailed response to all comments, with references to the tracked-changes version of the manuscript.

We hope that the revised version meets the Journal’s standards and can be accepted for publication.

Sincerely,

Felipe Dutra Rêgo

On behalf of all authors

Instituto René Rachou - Oswaldo Cruz Foundation

## Reviewer 2

Reviewer comments to the authors

Reviewer #2:

*a) Abstract - The abstract would benefit from:*

*• Clearer specification of the methodological approaches used for host identification and parasite confirmation.*

*• More precise wording regarding the strength of the phylogenetic support.*

*• Avoiding overstatement of novelty without robust comparative evidence.*

Authors’ comment: The abstract has been revised to improve precision and to better reflect the methodological and interpretative scope of the study. Specifically, the following adjustments were made: (1) a brief clarification on host identification was added (line 23). Given the word limit constraints, detailed diagnostic criteria were not included in the abstract but are provided in the Methods section; (2) the wording describing phylogenetic inference was adjusted to avoid overstatement, replacing “identified as” with the more conservative “placed within” which more accurately reflects the level of support provided by 18S rRNA and gGAPDH sequence data (lines 30-31), and (3) minor modifications were introduced to moderate the tone of the conclusions and avoid overstating novelty, while preserving the main findings and their relevance (lines 33-37).

*b) Originality and Importance of the Contribution - The originality claim is weakened by:*

*• Insufficient comparison with previously described isolates.*

*• Lack of detailed morphometric comparison with the original description by Votypka & Suková (2013).*

*• Limited discussion of prior regional reports of monoxenic trypanosomatids in fleas (e.g., reports from Minas Gerais).*

Authors’ comment: The manuscript has been revised to strengthen the contextualization of the findings and to highlight the contribution of the study to the current knowledge on trypanosomatid flagellates in fleas. Specifically, the following adjustments were made: (1) We performed a morphological comparison of our strain with the type strain described by of *B. campbelli* by Votýpka et al. (2013), but noted that the extremely concise nature of the original description renders such a comparison unreliable, whereas available molecular sequences clearly indicate that both belong to the same species (see Results and Discussion, lines 273-282). (2) Prior reports of trypanosomatids in fleas from Brazil, including studies conducted in Minas Gerais, have been mentioned (lines 333-338). We highlight that morphometric data present in one of these studies overlap with those estimated by us, suggesting similarity of the isolated parasites, although molecular evidence for this is lacking. (3) The overall wording of the manuscript has been adjusted to more accurately reflect the nature of the contribution.

*c) Methodology, Results, and Discussion:*

*1. Host Identification:*

*The identification of Ctenocephalides felis is not sufficiently documented.*

*• No detailed morphological description is provided.*

*• No indication of chemical clearing procedures for proper visualization of cephalic and comb structures.*

*• No molecular confirmation (e.g., COI barcoding) is reported.*

*• No explicit differentiation from Ctenocephalides canis or other related taxa.*

Authors’ comment: The manuscript has been revised to improve the documentation of host identification: (1) a representative figure has been added (Figure 1) showing diagnostic morphological characters used for flea identification, now also listed in the text; and (2) it has been clarified in Material and Methods that identification was performed on fresh specimens under stereomicroscopy using standard taxonomic keys (lines 99-102). Although chemical clearing and slide mounting can facilitate detailed morphological examination in arthropods, these procedures are not required for species-level identification in *Ctenocephalides*, where key diagnostic characters are readily visible in untreated specimens.

Molecular confirmation (COI barcoding) was not performed, as morphological identification remains the standard approach in studies involving *Ctenocephalides* spp., particularly in regions where closely related species can be reliably distinguished using well-established morphological traits. Moreover, specimen processing for parasite isolation (maceration and cultivation) was not compatible with clearing or molecular barcoding.

Species identification was conducted by Dr. Daniel Avelar, co-author of this study and curator of the Siphonaptera collection originally established by Prof. Pedro Marcos Linardi, ensuring taxonomic accuracy and consistency with established expertise in the group.

*2. Isolation Procedures and Parasite Detection - The manuscript does not provide adequate methodological details regarding:*

*• Time and conditions between flea capture and processing.*

*• Dissection procedures.*

*• Culture medium composition.*

*• Temperature conditions.*

*• Subculturing frequency.*

*• Contamination control measures.*

Authors’ comment: The manuscript has been revised to provide additional methodological details regarding parasite isolation and cultivation. Specifically, information has been added on (1) conditions between flea collection and processing, including transport under controlled temperature and humidity and same-day processing (lines 94-98); (2) clarification of the culture medium composition, explicitly defining the biphasic NNN/LIT medium (lines 107-109); (3) subculturing frequency (lines 110-111).

Contamination control measures, including antibiotic and antifungal supplementation, incubation conditions, and pre-incubation steps to reduce microbial load were already described in the previous version of the manuscript.

*3. Anatomical Localization – The anatomical localization of the parasite within the flea is not clearly described. Given that Blechomonas species are commonly associated with the hindgut surface, and the authors hypothesize the presence of a “uromonad” stage involved in attachment, the study should:*

*• Specify where parasites were observed.*

*• Provide microscopy images supporting localization.*

*• Compare findings with previous descriptions.*

Authors’ comment: The anatomical localization of the parasite within the flea was not assessed in the present study, as fleas were processed by whole-body maceration for culture-based isolation, and subsequent characterization was performed exclusively in culture. We acknowledge that information on parasite localization within the host would be valuable, especially given that it not available in the original description. However, addressing this question was beyond the scope of the present work.

The identification of “uromonads” as an attachment stage is based on observations of their behaviour in culture and comparisons with known attachment stages (e.g., haptomonads) described in other trypanosomatids, as well as previously reported hindgut-associated forms of *Blechomonas* spp. This interpretation has been revised in the manuscript to avoid overstatement.

We have also explicitly acknowledged the lack of anatomical localization as a limitation and clarified that future studies using dissected specimens and in situ approaches will be necessary to confirm the localization and attachment mechanisms of this species within the flea host (lines 258-263).

*4. Morphological Characterization and Taxonomic Context – Although morphometric measurements were performed, there is no structured comparison with the original description of Blechomonas campbelli.*

*This is particularly important because:*

*• Species in this genus exhibit overlapping morphological traits.*

*• Morphology alone is insufficient for reliable identification.*

*• Previous cases (e.g., reclassification of Leptomonas pulexsimulantis to Blechomonas) demonstrate the limitations of morphology-based taxonomy.*

Authors’ comment:

The aim of this study is not taxonomic revision or species delimitation, but the characterization of a cultured isolate and its biology. We agree that morphology alone (particularly at the light microscopy level) is insufficient for reliable species identification in trypanosomatids and that morphological traits may overlap among species. Species identification was therefore performed based on sequence data, while the morphological analysis was intended to expand the currently limited information available for *Blechomonas campbelli*, rather than to serve as a basis for taxonomic discrimination. The original description of this species contains only minimal morphological data, which limits the scope for a structured comparative analysis (see also response to comment 1b). In our study, the morphological analysis primarily aims to provide insights into the parasite’s life cycle, with appropriate acknowledgment of its limitations (see response to comment 3).

The manuscript has been revised to include a comparison of our isolate with the original description of *B. campbelli* and an earlier paper providing morphometric data on trypanosomatids from cat fleas (lines 283-294).

*5. Phylogenetic Analyses - The phylogenetic analysis would benefit from:*

*• Broader inclusion of representative isolates from multiple trypanosomatid genera.*

*Restricted sampling can artificially inflate bootstrap or posterior probability values due to reduced topological alternatives. Strong support values under limited sampling do not necessarily reflect true phylogenetic stability. Broader sampling would improve clade stability and strengthen species-level assignment.*

Authors’ comment: Our phylogenetic analysis was specifically designed to resolve relationships within a single genus (*Blechomonas*) rather than across multiple trypanosomatid genera, which has been already efficiently achieved by a phylogenomic analysis (Kostygov et al., 2024). To this end, we included all available sequences representing multiple species and strains within that genus, ensuring dense and representative ingroup sampling. Because this genus does not have a well-established close sister lineage at the genus or subfamily level and instead branches as sister to a large clade comprising most other trypanosomatid genera, we selected a limited set of representatives from this clade, along with a more distant taxon (*Trypanosoma*), for outgroup rooting. Expanding the sampling across this diverse clade would not increase resolution within the focal genus but would substantially increase dataset complexity and overall tree size without clear benefit for the question addressed here.

In this context, support values are driven primarily by ingroup sampling, which is comprehensive in our dataset, rather than by the breadth of representation of more distantly related taxa.

*6. Functional Evidence for Thermal Adaptation – The manuscript discusses thermotolerance but does not provide mechanistic or functional validation.*

*If heat tolerance is proposed as a biological trait, it should be supported by:*

*• Growth curves at multiple temperatures.*

*• Statistical comparison of proliferation rates.*

*• Gene expression assays (e.g., RT-qPCR) evaluating HSP70, HSP90, desaturases, and flagellar proteins.*

*• Normalization against housekeeping genes such as GAPDH.*

Authors’ comment: Our study does not aim to investigate the mechanisms of thermotolerance, but rather to assess temperature-dependent growth as one of the traits characterizing the strain. In this context, our data demonstrate a lack of thermotolerance, as evidenced by the absence of proliferation at 35 °C and reduced growth at 30 °C (Figure 2 and Supplementary Data - Sheet1). The additional experiments proposed (e.g., gene expression analyses of heat shock proteins) would address the molecular basis of thermotolerance and represent a distinct line of investigation beyond the scope of this work. We have clarified this point in the revised manuscript (lines 200-202).

The raw statistical data are provided in Supplementary Material - Sheet1.

# Reviewer report: REVIEWERS COMMENTS

## REVIEWER #1

No comments.

## REVIEWER #2

No comments.

## REVIEWER #3

No comments.
